# Supplementary material for: Bayesian refinement of protein structures and ensembles against SAXS data using molecular dynamics
Source: PLoS Comput Biol. 2017 Oct 18;13(10):e1005800. doi: 10.1371/journal.pcbi.1005800 (PMC5662244; doi:10.1371/journal.pcbi.1005800)
Supplement: S1 Archive — (BZ2) [file pcbi.1005800.s011.tar.bz2 › gromacs_4.62_bayesian_saxs_refi/share/html/online/mixed.html]

Getting started - Methanol+Water


| Getting started - Methanol+Water Main Table of Contents |

**VERSION 4.6  
Sat 19 Jan 2013**

---

## Methanol+Water

Now you are going to simulate 216 molecules of methanol and 216
molecules of water in a rectangular box (of 4.72 x 2.36 x 2.36 nm).
The molecules are completely demixed in the start conformation.

Change your directory to tutor/mixed :
  
  

|  |  |  |
| --- | --- | --- |
|  | cd tutor/mixed  \| |

  
Start by viewing the simulation box graphically:
  
  

|  |  |  |
| --- | --- | --- |
|  | rasmol mixed.pdb   \| |

  
Note that one side of the box only contains methanol while the other only
contains water.

Since all the neccesary files are available, we are going to,
preprocess all the input files to create a run input
(.tpr) file.
This run input file is the only input file for the
MD-program mdrun.
  
  

|  |  |  |
| --- | --- | --- |
|  | grompp -v   \| |

  
Now it's time to start the simulation of 1000 picoseconds. Since this will
take some time, it has to be started in the background, otherwise you will
not be able to log out without terminating the simulation.
  
  

|  |  |  |
| --- | --- | --- |
|  | nohup mdrun -v >& log &   \| |

  
After the MD simulation is finished (but even while it is still running),
it is possible to view the
trajectory with the ngmx program:
  
  

|  |  |  |
| --- | --- | --- |
|  | ngmx   \| |

  

When the program starts, you must select a group of atoms to view.
You can choose to select only one group, or both. If you select first methanol
and then rewind the trajectory and select water, you see how the mixing takes
place.

---

## Analysis of the simulation

1. First we will analyze the mixing process. We can compute the density
   of molecules, along the long axis of the simulation box, at different
   times in the simulation.
     
     

   |  |  |  |
   | --- | --- | --- |
   |  | g\_density -n index -o dens0 -b 0 -e 50 -d X   \| |

     
   Here the -b and -e options indicate begin and end of the
   analysis. When asked which groups to analyse you select two groups,
   MeOH and Water. Now do the same for four more stretches of 50 ps along the
   1000 ps trajectory (remember to change the name of the output file as well),
   e.g. with begin times 0, 240, 480, 720, 950). And view all the output files
   at once (if you used different names, replace the ones below with those):
     
     

   |  |  |  |
   | --- | --- | --- |
   |  | xmgrace -nxy dens0.xvg -nxy dens240.xvg -nxy dens480.xvg -nxy dens720.xvg -nxy dens950.xvg -legend load   \| |

     
   Explain the results. When (at which timepoint)
   do you consider the system completely mixed?
2. Calculate a radial distribution function of the oxygen atoms
   around oxygen atoms. The index file index.ndx now contains multiple groups.
   Select oxygen (containing both the water oxygen and the methanol oxygen).
     
     

   |  |  |  |
   | --- | --- | --- |
   |  | g\_rdf -n index -o rdf-oo.xvg -b 900   \| |

     
   The program will ask you for how many groups you want the calculate
   the RDF, answer 1 (and select oxygen and oxygen). We start at 900 ps
   in order to only use the completely mixed system.
   Now, view the output graph.
     
     

   |  |  |  |
   | --- | --- | --- |
   |  | xmgrace rdf-oo.xvg   \| |

     
   Which shows you the radial distribution function for oxygen-oxygen in the
   mixture. Now do the same thing using the methyl group as reference and
   as target (and use e.g. rdf-mm.xvg as output file name). Do not
   forget the -b 900 option to g\_rdf. View all
   the graphs together:
     
     

   |  |  |  |
   | --- | --- | --- |
   |  | xmgrace rdf-oo.xvg ../methanol/rdf-oo.xvg ../water/rdf.xvg -legend load   \| |

     
   The xmgrace program will display three different graphs.
   Compare the resulting graphs. Explain the differences and the
   similarities.  
   Do the same analysis for the Me-Me RDF in the mixture and in pure methanol.
   Compare the resulting graphs. Explain the differences and the
   similarities.
3. We can also do a direct analysis of the number of hydrogen bonds in
   methanol, based on O-O distance and O-H ... O angle.
     
     

   |  |  |  |
   | --- | --- | --- |
   |  | g\_hbond   \| |

     
   Select twice 0, when asked. Check the output with
     
     

   |  |  |  |
   | --- | --- | --- |
   |  | xmgrace hbnum.xvg   \| |

     
   What is the number of hydrogen bonds per
   molecule? Compare the results to
   those from pure water and from pure methanol. Does the total
   number of hydrogen bonds change during the mixing process?
4. As a further test of the simulation we will compute the self
   diffusion constant of Methanol and water in the mixed state.
     
     

   |  |  |  |
   | --- | --- | --- |
   |  | g\_msd -n index -b 900   \| |

     
   (Run it twice, first selecting Me1 and then OW). View the output
     
     

   |  |  |  |
   | --- | --- | --- |
   |  | xmgrace msd.xvg   \| |

     
   Check that the graph is roughly linear. The g\_msd program also
   computes the diffusion constant D for you.
   Compare the result to pure water and pure methanol.
   Is it as you would expect?

---


### Go to the next step


---
